# Integrated Remediation Processes Toward Heavy Metal-Contaminated Environment

**DOI:** 10.3390/toxics13070557

**Published:** 2025-06-30

**Authors:** Luhua Jiang, Liang Hu, Zhigang Yu

**Affiliations:** 1 School of Minerals Processing and Bioengineering, Central South University, Changsha 410083, China; huliang2018@csu.edu.cn; 2 Key Laboratory of Biometallurgy of Ministry of Education, Central South University, Changsha 410083, China; 3 Australian Centre for Water and Environmental Biotechnology, St Lucia, QLD 4072, Australia; zhigang.yu@uq.edu.au

## 1. Introduction

This Editorial introduces the Special Issue titled “Integrated Remediation Processes toward Heavy Metal-Contaminated Environment”. With the development of urban industrialization and the utilization of mineral resources, significant amounts of heavy metals have been released into aquatic ecosystems, agricultural soils, and the atmosphere, posing a serious threat to human health. Given the severe hazards associated with heavy metals, there is growing interest in exploring remediation technologies from a more comprehensive perspective [1]. Concurrently, under the national dual carbon target, researchers worldwide are striving to harness integrated technologies to treat contaminated water, soil, and even groundwater. It is expected that the dual carbon target and successful heavy metal remediation can be achieved concurrently by removing or stabilizing heavy metals in the environment, thereby reducing their ecological risks [2].

This Special Issue features nine articles¸ including seven research papers and two reviews. The research papers focus on comprehensive remediation strategies, underlying mechanisms, and impact assessments related to environmental heavy metal contamination. The reviews focus on recent advances, mechanisms of action, and future prospects of remediation agents for heavy metal pollution. These articles enhance our understanding of heavy metal pollution control and support its advancement toward greater efficiency, resource recovery, and ecological safety.

## 2. An Overview of Published Articles

The development of novel approaches for the efficient and safe remediation of heavy metal contamination is both imperative and challenging. Hence, Nie et al. (Contribution 1) provide a comprehensive analysis of recent advances in soil amendments for heavy metal-contaminated soil remediation, with a focused examination of natural, synthetic, natural–synthetic copolymer, and biological amendments. Through systematic comparison of their remediation mechanisms and effectiveness, this study offers a thorough evaluation of their impacts on soil physicochemical properties, leachable heavy metal concentrations, and microbial community structure.

This Special Issue presents five articles investigating heavy metal removal in polluted environments, three articles focusing on materials and their coupling technologies, and two articles concentrating on microbial approaches. Gertsen et al. (Contribution 2) synthesized organoclays based on bentonite using various amphoteric and nonionic surfactants and evaluated their performance as efficient sorbents for lead ions. Among them, the organoclay-based bentonite with alkyl polyglucoside exhibited the optimal performance, achieving a maximum adsorption capacity of 1.49 ± 0.05 mmol/g. These novel organoclays demonstrate significant potential for remediating lead-contaminated water and soil. Zhang et al. (Contribution 3) proposed a novel modified pulse electrochemical treatment (PECT) method that integrates biochar as a permeable reactive barrier into the PECT system, with acetate incorporation in the catholyte. This research revealed that the biochar-coupled PECT system with optimized pulse gradients achieved high lead removal efficiency while reducing energy consumption and treatment time. Notably, biochar has been widely applied for heavy metal remediation due to its exceptional adsorption capacity and persistent free radical (PFR) content. However, the environmental behavior and potential risks associated with both biochar and its associated PFRs warrant careful consideration. Alfei et al. (Contribution 4) conducted a systematic investigation into the application potential, mechanisms of action, and associated risks of biochar and its contained PFRs in environmental remediation. This review highlights that biochar—particularly the PFRs generated during its production via pyrolysis—may represent a double-edged sword in environmental applications. Consequently, comprehensive investigations into the ecological effects of biochar and the development of targeted mitigation strategies are critically needed.

For microbial approaches, Zhuang et al. (Contribution 5) analyzed the behavior and mechanism of sulfate-reducing bacteria *Desulfovibrio desulfuricans* in precipitating antimony from wastewater. The study demonstrated that SRB cells achieved antimony immobilization through a three-step process—adsorption, reduction, and sulfidation—on their surface, underscoring the coprecipitation role of phosphorus-containing groups. It also provided a theoretical foundation and technical parameters for SRB-based remediation of antimony-contaminated wastewater, particularly suitable for treating mining wastewater. Additionally, Hao et al. (Contribution 6) explored the impact of bioreactor scale-up cultivation on microbial succession in mixotrophic acidophiles from an industrial-scale perspective, alongside its application in remediating Cd-contaminated soil. Their study revealed that bioreactor scale-up drives pH reduction by altering bacterial communities (but not fungi), thereby indirectly enhancing cadmium (Cd) removal efficiency. They also identified 10 m^3^ as the critical scale for microbial community and functional shifts.

Acid mine drainage is one of the major sources of heavy metals in contaminated environments. Therefore, the safe treatment and resource utilization of waste rock and tailings are effective methods for controlling heavy metal pollution. Zhang et al. (Contribution 7) developed a cooperative leaching system (Fe_2_(SO_4_)_3_-O_3_) to investigate the oxidative dissolution of the waste sulfides, achieving a zinc extraction efficiency of 97.8% under optimal conditions. Guo et al. (contribution 8) investigated the leaching behavior of As and Pb in lead–zinc mining waste rock under mine drainage and rainwater conditions. The study found that As and Pb in mine waste mainly exist in sulfides (e.g., arsenopyrite, pyrite, galena), with smaller amounts adsorbed onto clays. Acidic conditions enhanced As/Pb release, while alkaline conditions increased arsenate mobility. The sulfide content governs the waste’s acid-generating potential and associated environmental risks. This study elucidated the mechanisms by which soil/water contamination occurs in lead–zinc mining areas, providing a scientific basis for pollution prevention and control. Moreover, in this Special Issue, Li et al. (contribution 9) highlighted the use of mathematical estimation of endogenous proline as a bioindicator for assessing the stress response of rice plants to trivalent chromium under varying nitrogenous conditions. The study showed that plants alleviate Cr(III) toxicity by regulating proline levels across nitrogen regimes. The proline biomarker system, when combined with MBMM, facilitates nitrogen source assessment and supports the selection of optimal remediation strategies.

## 3. Conclusions

Continuously exploring comprehensive remediation processes for heavy metal-contaminated environments under the dual carbon goals—including developing novel heavy metal removal methods, controlling heavy metal migration at the source, and mitigating ecological impacts—is a critical research direction requiring in-depth investigation. The articles in this Special Issue offer multidimensional insights into the mechanisms, application potential, and risk management of environmental heavy metal remediation technologies, offering scientific support for the development of an eco-friendly heavy metal pollution control system.

## Data Availability

Not applicable.
